# Depression and anxiety among psychiatry residents

**DOI:** 10.1192/j.eurpsy.2022.1447

**Published:** 2022-09-01

**Authors:** N. Bouattour, W. Bouattour, M. Abdelkefi, N. Messedi, F. Charfeddine, L. Aribi, J. Aloulou

**Affiliations:** 1 Hedi Chaker University Hospital Ain road 0.5 Km, Psychiatry B, Sfax, Tunisia; 2 university hospital center Hedi Chaker Sfax, Psychiatry B, sfax, Tunisia; 3 Hedi Chaker University Hospital, Psychiatry ‘b’ Department, Sfax, Tunisia; 4 Hedi chaker, Hospital, Sfax, Tunisia; 5 Hedi Chaker University Hospital, Psychiatry “b” Department, Sfax, Tunisia

**Keywords:** Depression, Anxiety, Training residents in psychiatry, HADS

## Abstract

**Introduction:**

Residency training has been reported as being stressful which may lead to different mental disorders.

**Objectives:**

To study the prevalence and associated factors of anxiety and depression symptoms among psychiatry residents.

**Methods:**

A cross-sectional study was conducted through an online survey among psychiatry residents. Participants completed an anonymous self-administered questionnaire and the HADS questionnaire for screening anxiety and depression.

**Results:**

Forty responses were collected.The average age of the sample was 28.08 ± 2.43 and the sex-ratio (F/M) was 0.875. Eleven participants were married. Eight residents were smokers. The prevalence of alcohol use and cannabis use was 22.5 % and 5% respectively. Half of participants were first year residents and near three-quarter of them (72.5%) declared working in poor conditions. A considerable proportion of participants had symptoms of anxiety and depression. The prevalence of anxiety case and depression case was 52.5% and 47.5% respectively. The prevalence of Anxiety symptoms and depression symptoms was significantly higher in female participants (p = 0.017, p=0.034 respectively). Poor conditions of the workplace were significantly associated with depression symptoms (p=0.004).

**Conclusions:**

Training residents in psychiatry showed high rates of anxiety and depression symptoms. Screening and early management of these psychiatric manifestations is necessary. In addition, improving working conditions would upgrade their training and quality of life.

**Disclosure:**

No significant relationships.

